# Supplementary material for: Retrotranspositional landscape of Asian rice revealed by 3000 genomes
Source: Nat Commun. 2019 Jan 3;10:24. doi: 10.1038/s41467-018-07974-5 (PMC6318337; doi:10.1038/s41467-018-07974-5)
Supplement: Supplementary file 1 — Supplementary Information [file 41467_2018_7974_MOESM1_ESM.docx]

**Retrotranspositional landscape of Asian rice revealed by 3000 genomes**

Carpentier *et al.*


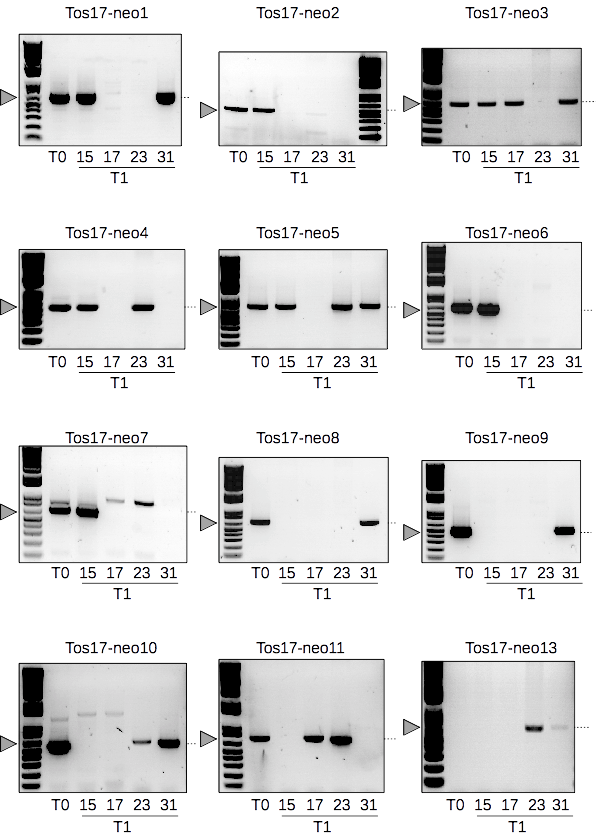


**Supplementary Figure 1**. Gels PCR Validation of Tos17 insertions.

A set of independently identified Tos17 insertions in a pedigree of four rice plants has been used as validation data for trackposon. T0 has been regenerated from callus after transformation by A. tumefasciens, conditions that allow Tos17 mobilization. T1-15, T1-17, T1-23 and T1-31 were obtained by selfing of the T0. After Illumina sequencing, reads have been mapped against the reference genome of Oryza sativa cv. nipponbare. Based on the position of its unique active copy, new insertions loci of Tos17 could be identified by visual analysis in a browser (IGV), of the discordantly aligned pairs, one mate of which matches the native TE copy : the discordant mates indicated the insertion position of the new copy.

Agarose gels obtained after PCR. The arrow and the dashed line indicate the position of the fragments of the expected size. Other bands are the result of random non specific amplifications. Size markers are at the extreme left or right of the gels. Colors were inverted for readability.


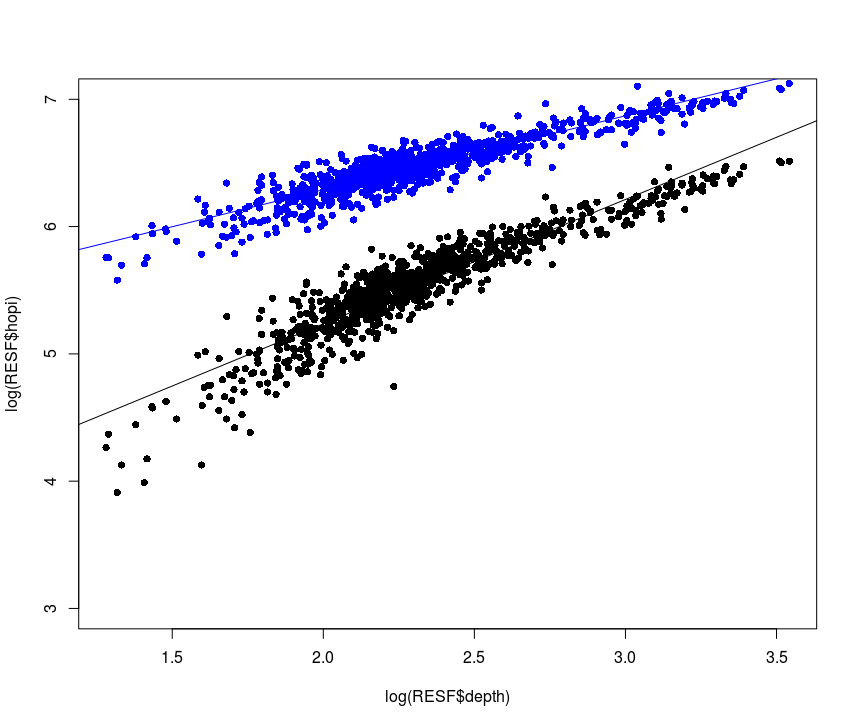


**Supplementary Figure 2**. The effect of the third step (threshold 2) on TIP detection.

In the TRACKPOSON pipeline, the third step was added to decrease the number of false negative TE insertions.In x axis, the depth coverage for the 3000 genomes was represented in log scale, and in y axis the number of Hopi insertions also in log scale.

In black, the result only the first pass at a threshold of 5 and in blue the result with the additional pass at a the threshold of 2. The lines correpond to the linear regression for the 2 representations.


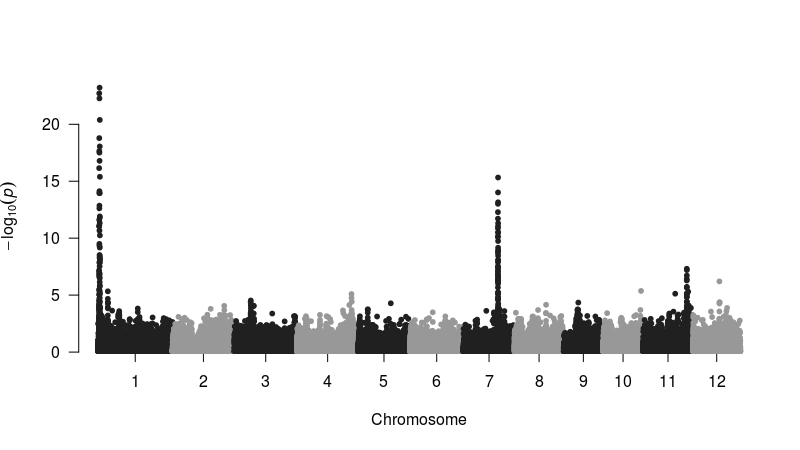

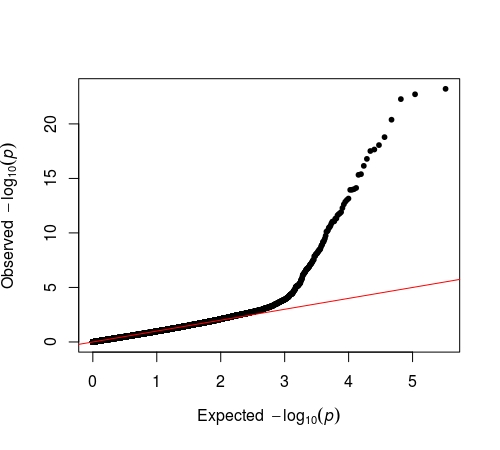


| TE family | GWAS interval | TE overlapping or candidate genes | % mappability |
| --- | --- | --- | --- |
| Tos17 | Chr1: 734611-1063997 | Overlap with Tos17 | 66 |
| Tos17 | Chr7: 19995068-20201905 | Overlap with Tos17 | 72 |
| Tos17 | Chr11:25393543-25431911 | No Tos17 | 61 |

**Supplementary Figure 3.** GWAS results for *Tos17* retrotransposons family:

The Manhattan plot represents the association peaks. A Qqplot is also provided.

The table provides the exact localization of the peak region, together with the percentage of mappability of the region, computed using the method described in the manuscript.


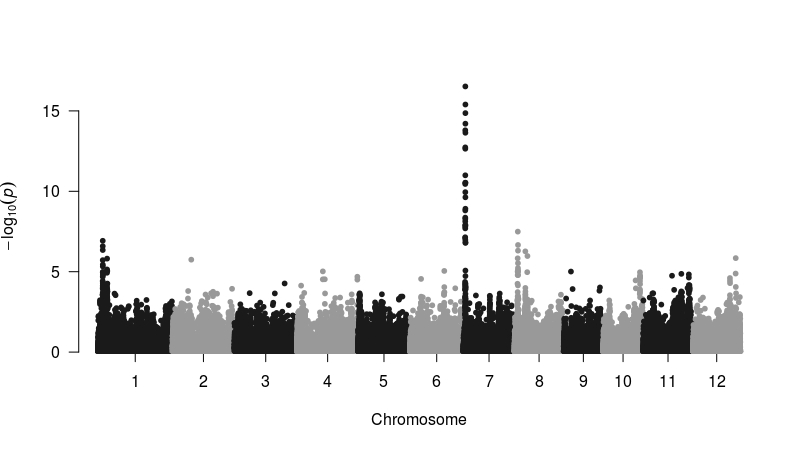


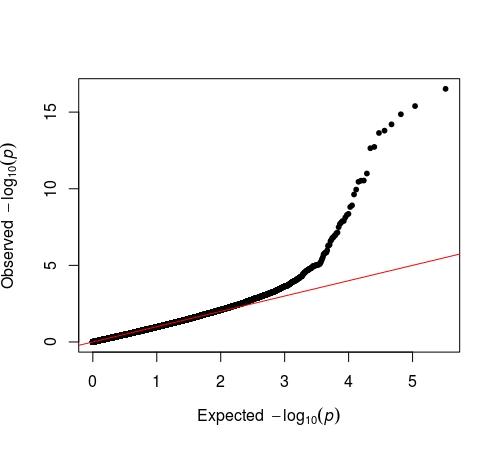


| TE family | GWAS interval | Candidate TE or genes | % mappability |
| --- | --- | --- | --- |
| Karma | Chr1: 2761585-2763810 | Overlap with karma copy | 95 |
| Karma | Chr7: 1043983-1159959 | Overlap with karma copy | 87 |
| Karma | Chr8: 1870881-1988117 | Overlap with karma copy | 28.5 |

**Supplementary Figure 4.** GWAS results for *Karma* retrotransposons family:

The Manhattan plot represents the association peaks. A Qqplot is also provided.

The table provides the exact localization of the peak region, together with the percentage of mappability of the region, computed using the method described in the manuscript.


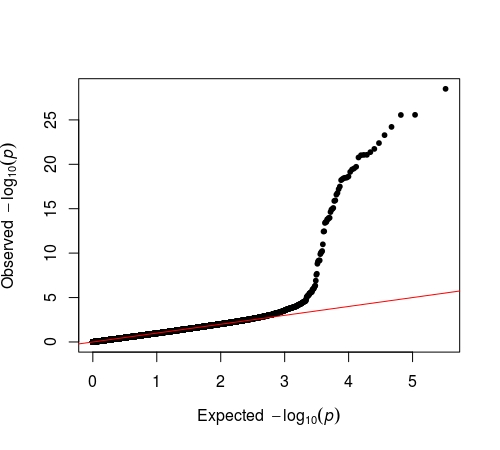

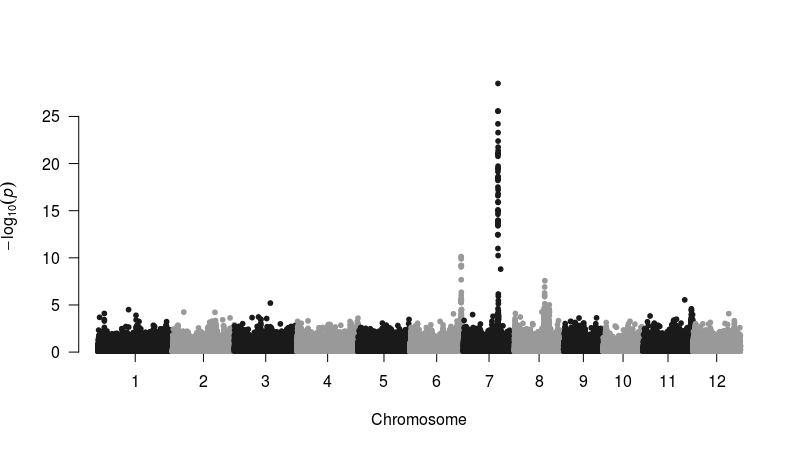


| TE family | GWAS interval | Candidate TE or genes | % mappability |
| --- | --- | --- | --- |
| Fam90 | Chr6: 29839249-29948887 | Overlap with Fam90 copy | 64 |
| Fam90 | Chr7: 19999141-20110067 | Overlap with Fam90 copy | 72F |
| Fam90 | Chr8: 17428187-17558707 | Overlap with Fam90 copy | 58 |

**Supplementary Figure 5.** GWAS results for *Fam90* retrotransposons family:

The Manhattan plot represents the association peaks. A Qqplot is also provided.

The table provides the exact localization of the peak region, together with the percentage of mappability of the region, computed using the method described in the manuscript.


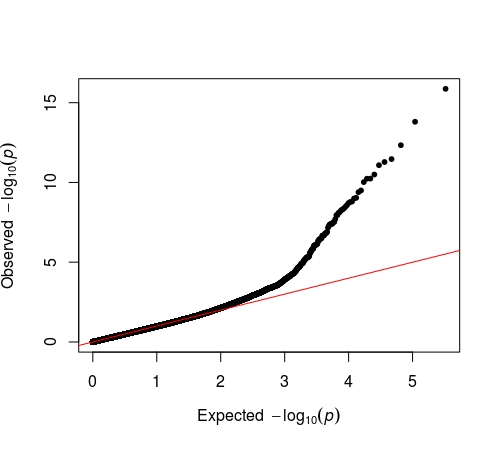

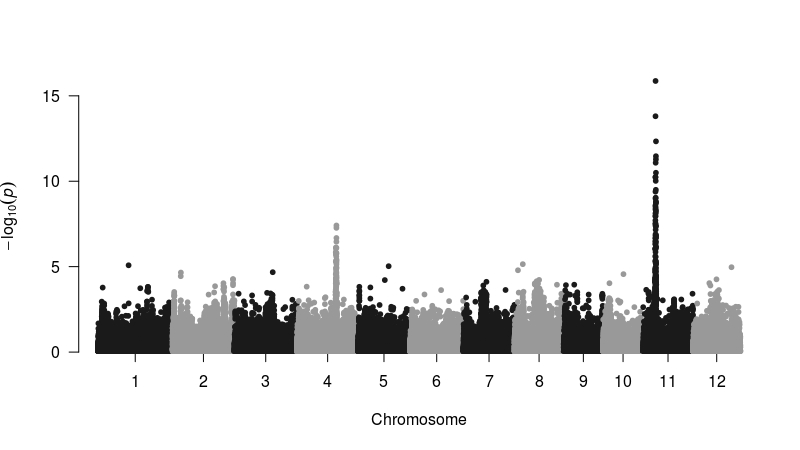


| TE family | GWAS interval | Candidate TE or genes | % mappability |
| --- | --- | --- | --- |
| Fam124 | Chr4: 22837141-22848622 | Overlap with Fam 124 | 87 |
| Fam124 | Chr11: 7012603-7530068 | No overlap with Fam 124 | *57* |

**Supplementary Figure 6.** GWAS results for *Fam124* retrotransposons family:

The Manhattan plot represents the association peaks. A Qqplot is also provided.

The table provides the exact localization of the peak region, together with the percentage of mappability of the region, computed using the method described in the manuscript.


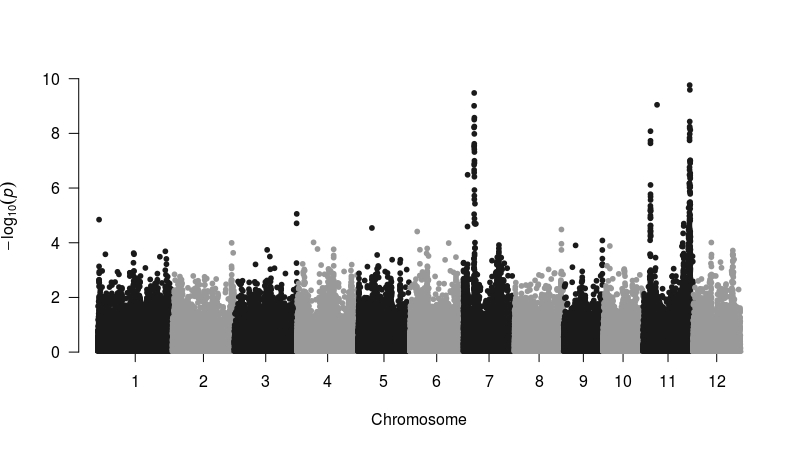

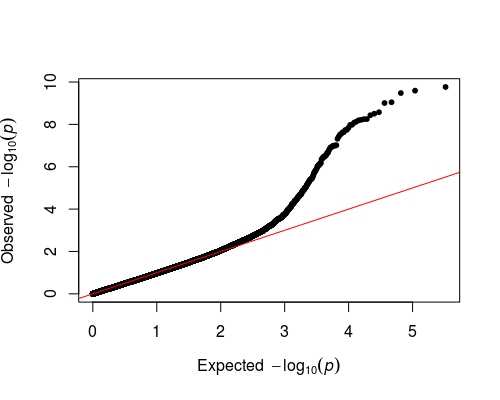


| TE family | GWAS interval | Candidate TE or genes | % mappability |
| --- | --- | --- | --- |
| Fam106 | Chr7: 6170573-6363341 | Overlap with fam106 copy | 41 |
| Fam106 | Chr11: 4316562-4375485 | Overlap with fam106 copy | 67 |
| Fam106 | Chr11: 27008905-27445698 | Overlap with fam106 copy | 62 |

**Supplementary Figure 7.** GWAS results for *Fam106* retrotransposons family:

The Manhattan plot represents the association peaks. A Qqplot is also provided.

The table provides the exact localization of the peak region, together with the percentage of mappability of the region, computed using the method described in the manuscript.


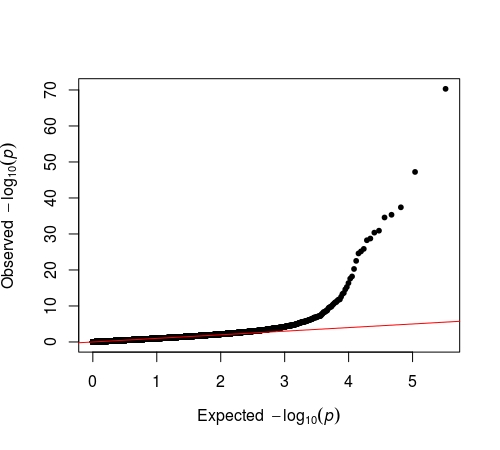

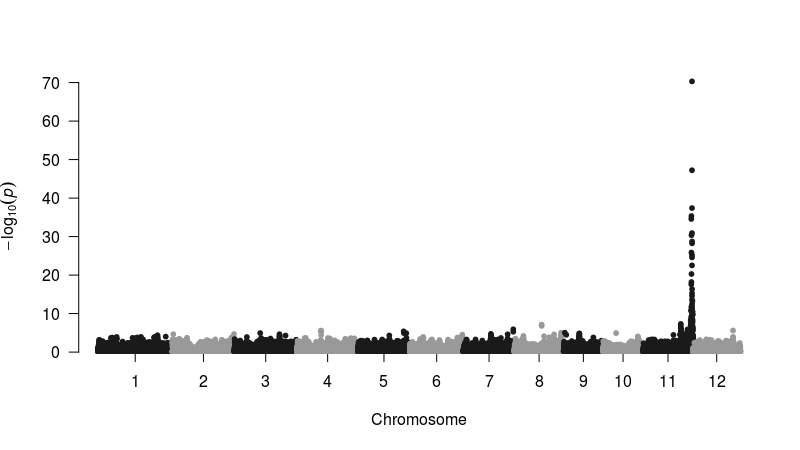


| TE family | GWAS interval | Candidate TE or genes | % mappability |
| --- | --- | --- | --- |
| Lullaby | Chr11: 28186914-28477992 | Overlap with lullaby copy | 68 |

**Supplementary Figure 8.** GWAS results for *Lulabby* retrotransposons family:

The Manhattan plot represents the association peaks. A Qqplot is also provided.

The table provides the exact localization of the peak region, together with the percentage of mappability of the region, computed using the method described in the manuscript.


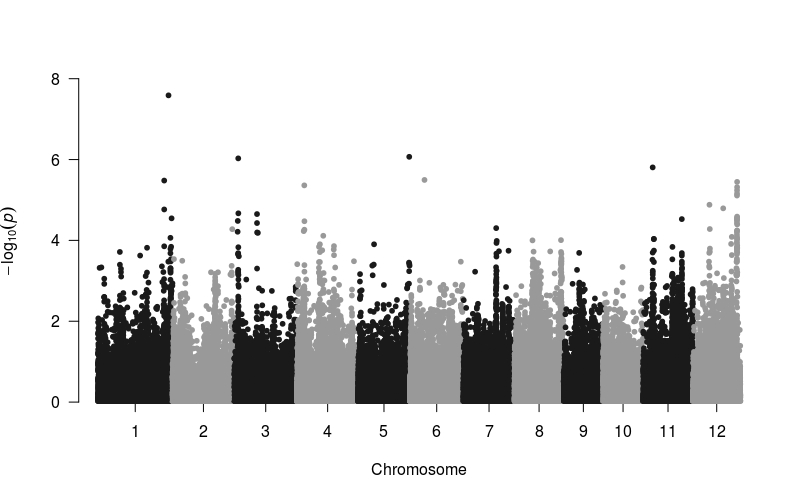

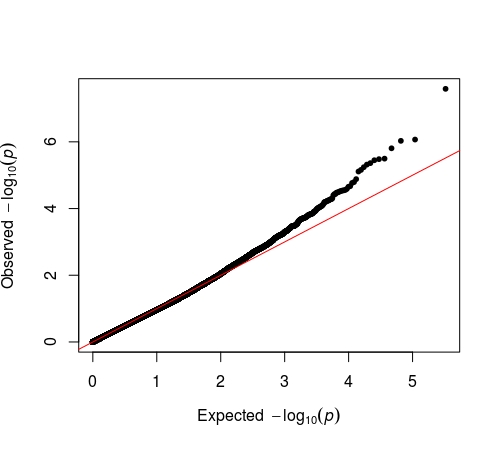


| TE family | GWAS interval | Candidate TE or genes | Mappability |
| --- | --- | --- | --- |
| Dasheng | Chr1:40946066 | Overlap with dasheng | 76 |
| Dasheng | Chr3:2251217-2312182 | Overlap with dasheng | 58 |

**Supplementary Figure 9.** GWAS results for *Dasheng* retrotransposons family:

The Manhattan plot represents the association peaks. A Qqplot is also provided.

The table provides the exact localization of the peak region, together with the percentage of mappability of the region, computed using the method described in the manuscript.


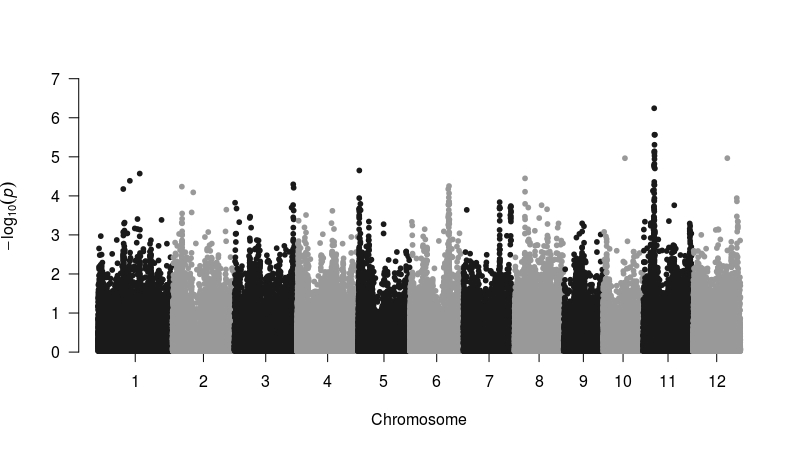

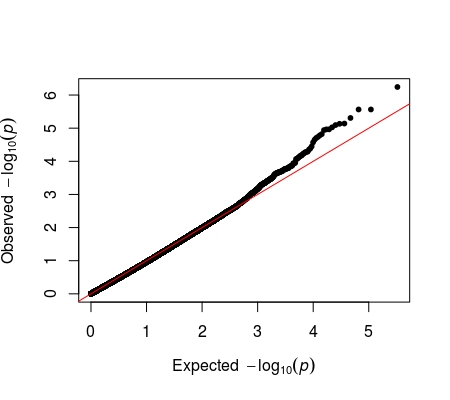


| TE family | GWAS interval | Candidate TE or genes | % mappability |
| --- | --- | --- | --- |
| Fam89 | Chr11: 6481747-6602990 | No overlap with Fam89 | 56 |

**Supplementary Figure 10.** GWAS results for *Fam89* retrotransposons family:

The Manhattan plot represents the association peaks. A Qqplot is also provided.

The table provides the exact localization of the peak region, together with the percentage of mappability of the region, computed using the method described in the manuscript.


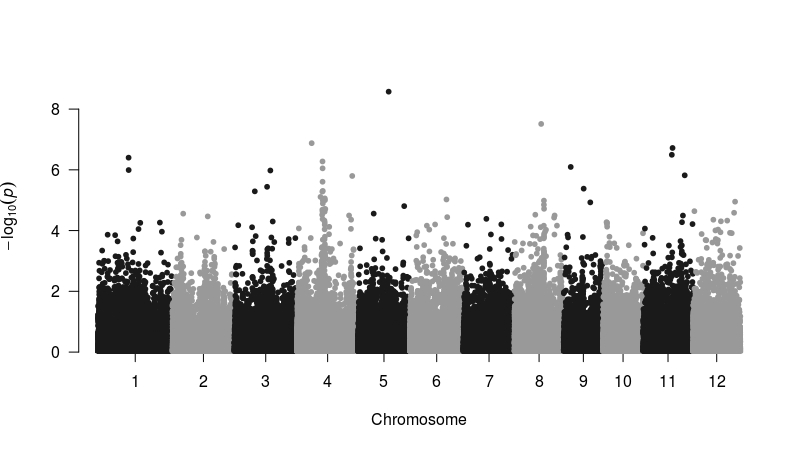

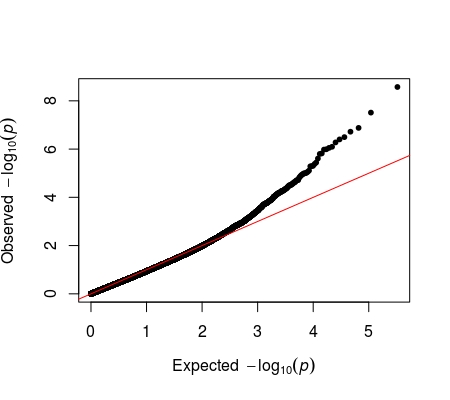


| TE family | GWAS interval | Candidate TE or genes | % mappability |
| --- | --- | --- | --- |
| Houba | Chr01: 17769540-17811498 | Centromere no overlap with Houba | 47 |
| Houba | Chr04: 14769176-14819944 | No overlap with Houba | 56 |

**Supplementary Figure 11.** GWAS results for *Houba* retrotransposons family:

The Manhattan plot represents the association peaks. A Qqplot is also provided.

The table provides the exact localization of the peak region, together with the percentage of mappability of the region, computed using the method described in the manuscript.


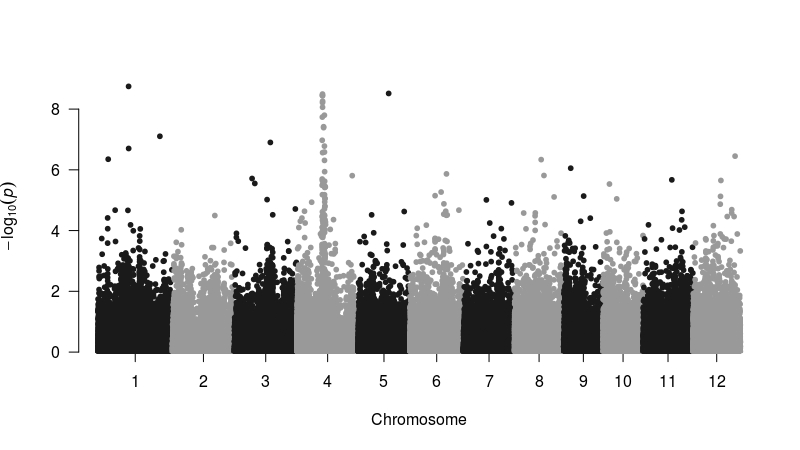

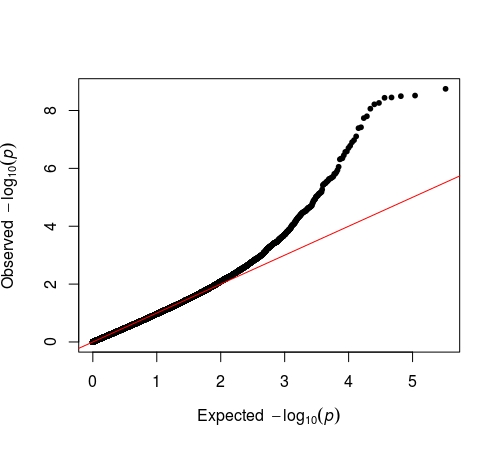


| TE family | GWAS interval | Candidate TE or genes | % mappability |
| --- | --- | --- | --- |
| Fam86 | Chr01: 17769540-17811498 | No overlap with Fam86 | 47 |
| Fam86 | Chr4:14599121-14827052 | No overlap with Fam86 | 53 |

**Supplementary Figure 12.** GWAS results for *Fam86* retrotransposons family:

The Manhattan plot represents the association peaks. A Qqplot is also provided.

The table provides the exact localization of the peak region, together with the percentage of mappability of the region, computed using the method described in the manuscript.


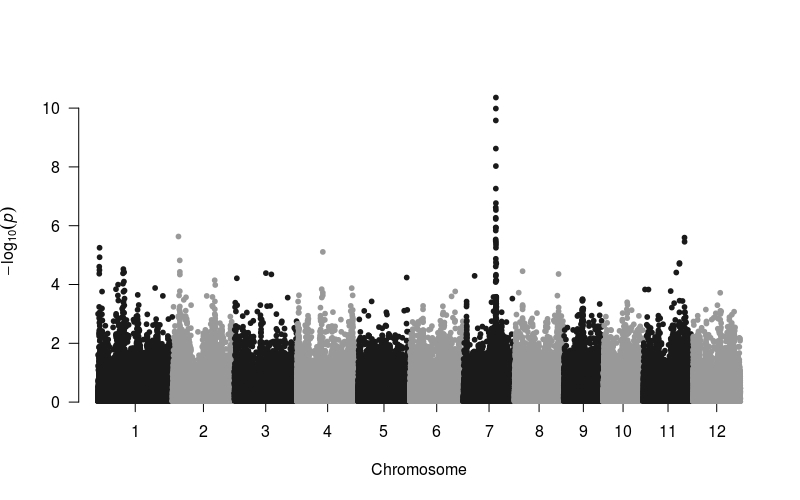

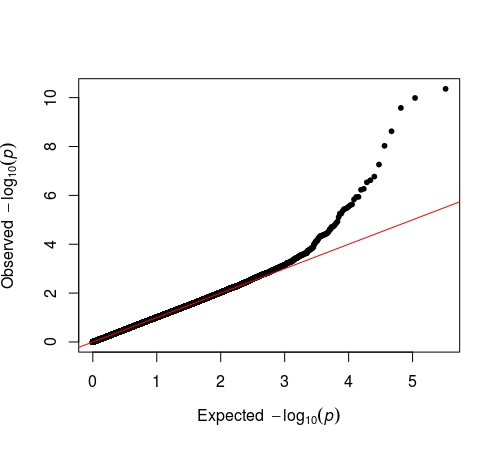


| TE family | GWAS interval | Candidate TE or genes | % mappability |
| --- | --- | --- | --- |
| rn304 | Chr07: 18726085-18820090 | Overlap with rn304 copy | 55 |

**Supplementary Figure 13.** GWAS results for *Rn304* retrotransposons family:

The Manhattan plot represents the association peaks. A Qqplot is also provided.

The table provides the exact localization of the peak region, together with the percentage of mappability of the region, computed using the method described in the manuscript.


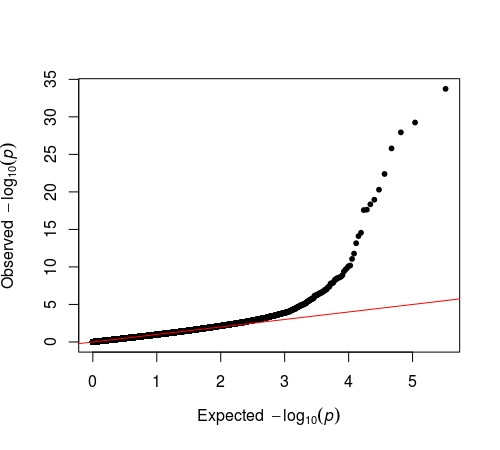


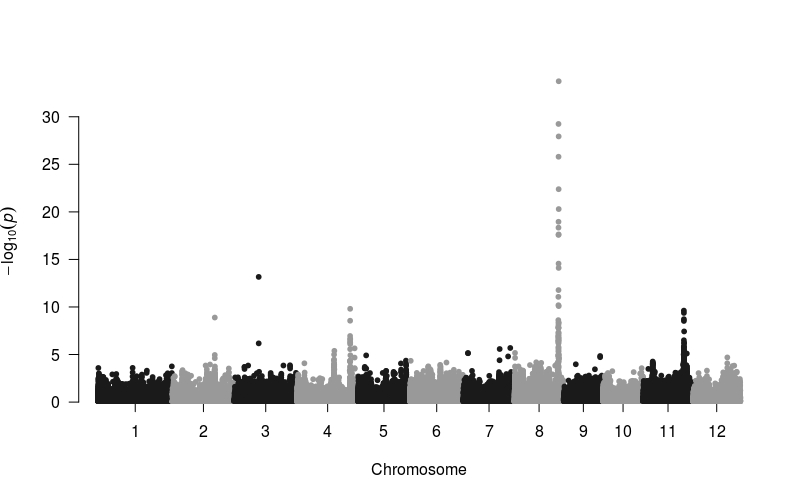


| TE family | GWAS interval | Candidate TE or genes | % mappability |
| --- | --- | --- | --- |
| Scaff3 | Chr04: 30805389-30819182 | Overlap with scaff3 copy | 49 |
| Scaff3 | Chr08: 25480885-25631114 | Overlap with scaff3 copy | 55 |
| Scaff3 | Chr11: 23710053-23731730 | Overlap with scqff3 copy | 31 |

**Supplementary Figure 14.** GWAS results for *Scaff3* retrotransposons family:

The Manhattan plot represents the association peaks. A Qqplot is also provided.

The table provides the exact localization of the peak region, together with the percentage of mappability of the region, computed using the method described in the manuscript.

**Supplementary Table 1.** The summary of *Tos17* insertions results


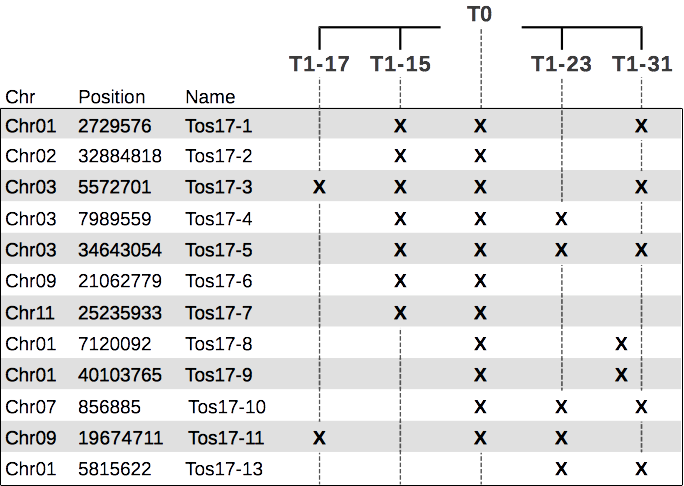


The summary of results, where a X indicates that the insertion is present.

All these insertions have been confirmed by PCR (see Supplementary Figure 1).

**Supplementary Table 2.** The list of primers for *Tos17* insertions validation


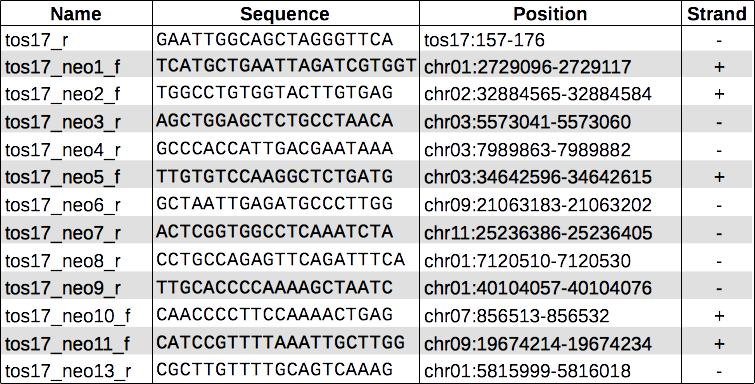


The list of name, sequence, position and strand of each primer used for validation.The first in the list, tos17_r, has been used with each other primer to check for the presence of each insertion

**Supplementary Table 3**. Gypsy and Copia TIPs localisation

|  | 1st quantile | Median | Mean | 3th quantile |
| --- | --- | --- | --- | --- |
| *Copia* | 182 nt | 11,296 nt | 22,888 nt | 32,064 nt |
| *Gypsy* | 432 nt | 11,665 nt | 23,459 nt | 32,733 nt |

We determinated for all the TIPs, the distance in nucleotides with the closest gene

(see Methods). We performed a Whelch t-test between these two dsitributions with

p.value of 1.6e-07. The Copia and Gypsy distribution are significativly different and

the Copia TIPs are closer to the genes.
